# Supplementary material for: A Global Screen for Assembly State Changes of the Mitotic Proteome by SEC-SWATH-MS
Source: Cell Syst. 2020 Feb 26;10(2):133–155.e6. doi: 10.1016/j.cels.2020.01.001 (PMC7042714; doi:10.1016/j.cels.2020.01.001)

P00558 | PGK1_HUMAN | PGK1 PGKA MIG10 OK/SW−cl.110
Monomer MW [kDa]: 44.615 Monomer expected elution fraction: 47

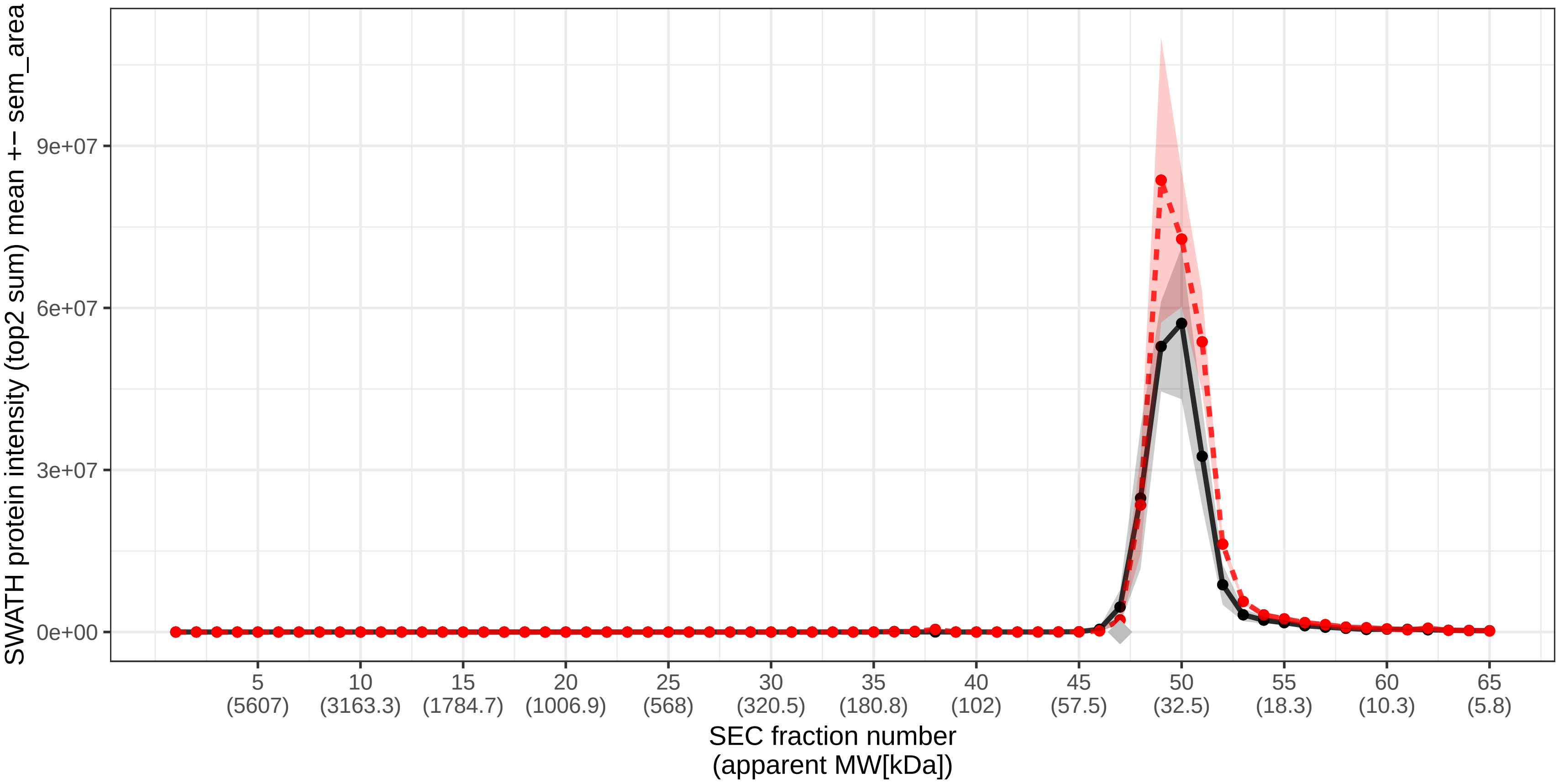

Supplement: Data S1. SEC-SWATH-MS Protein Chromatograms, Related to Figure 1 [file mmc6.zip › SECchrom_P00558_PGK1_HUMAN_PGK1_PGKA_MIG10_OK_SW-cl.110.pdf]
